# Impact of acute total occlusion of the culprit artery on outcome in NSTEMI based on the results of a large national registry

**DOI:** 10.1186/s12872-021-02099-y

**Published:** 2021-06-14

**Authors:** Michał Terlecki, Wiktoria Wojciechowska, Dariusz Dudek, Zbigniew Siudak, Krzysztof Plens, Tomasz J. Guzik, Tomasz Drożdż, Jan Pęksa, Stanisław Bartuś, Wojciech Wojakowski, Marek Grygier, Marek Rajzer

**Affiliations:** 1 Jagiellonian University Medical College, 1st Department of Cardiology, Interventional Electrocardiology and Arterial Hypertension, Jakubowskiego St. 2, 30-688 Kraków, Poland; 2 Jagiellonian University Medical College, 2nd Department of Cardiology, Kraków, Poland; 3 Faculty of Medicine and Health Sciences, Jan Kochanowski University, Kielce, Poland; 4 KCRI, Kraków, Poland; 5 Jagiellonian University Medical College, Department of Internal and Agricultural Medicine, Kraków, Poland; 6 Department of Cardiology and Structural Heart Diseases, Medical University of Silesia, Katowice, Poland; 7 1St Department of Cardiology, Poznan University of Medical Sciences, Poznan, Poland

**Keywords:** Myocardial infarction, STEMI, NSTEMI, Coronary angiography, Total artery occlusion

## Abstract

**Background:**

The impact of acute total occlusion (TO) of the culprit artery in non-ST-segment elevation myocardial infarction (NSTEMI) is not fully established. We aimed to evaluate the clinical and angiographic phenotype and outcome of NSTEMI patients with TO (NSTEMI_TO_) compared to NSTEMI patients without TO (NSTEMI_NTO_) and those with ST-segment elevation and TO (STEMI_TO_).

**Methods:**

Demographic, clinical and procedure-related data of patients with acute myocardial infarction who underwent percutaneous coronary intervention (PCI) between 2014 and 2017 from the Polish National Registry were analysed.

**Results:**

We evaluated 131,729 patients: NSTEMI_NTO_ (n = 65,206), NSTEMI_TO_ (n = 16,209) and STEMI_TO_ (n = 50,314). The NSTEMI_TO_ group had intermediate results compared to the NSTEMI_NTO_ and STEMI_TO_ groups regarding mean age (68.78 ± 11.39 vs 65.98 ± 11.61 vs 64.86 ± 12.04 (years), p < 0.0001), Killip class IV on admission (1.69 vs 2.48 vs 5.03 (%), p < 0.0001), cardiac arrest before admission (2.19 vs 3.09 vs 6.02 (%), p < 0.0001) and death during PCI (0.43 vs 0.97 vs 1.76 (%), p < 0.0001)—for NSTEMI_NTO_, NSTEMI_TO_ and STEMI_TO_, respectively. However, we noticed that the NSTEMI_TO_ group had the longest time from pain to first medical contact (median 4.0 vs 5.0 vs 2.0 (hours), p < 0.0001) and the lowest frequency of TIMI flow grade 3 after PCI (88.61 vs 83.36 vs 95.57 (%), p < 0.0001) and that the left circumflex artery (LCx) was most often the culprit lesion (14.09 vs 35.86 vs 25.42 (%), p < 0.0001).

**Conclusions:**

The NSTEMI_TO_ group clearly differed from the NSTEMI_NTO_ group. NSTEMI_TO_ appears to be an intermediate condition between NSTEMI_NTO_ and STEMI_TO_, although NSTEMI_TO_ patients have the longest time delay to and the worst result of PCI, which can be explained by the location of the culprit lesion in the LCx.

## Background

According to the European Society of Cardiology (ESC) guidelines, patients with myocardial infarction (MI) and ST-segment elevation (STEMI) are eligible for emergency reperfusion therapy, whereas those with non-ST-segment elevation MI (NSTEMI) require further risk stratification; thus, the qualification for invasive diagnosis and treatment is delayed [1]. The STEMI-NSTEMI paradigm is based on the observation that ST-segment elevation (STE) on the electrocardiogram (ECG) in the majority of patients with MI is associated with acute total occlusion (TO) of the infarct-related artery (IRA), while subtotal IRA occlusion leads mostly to ST-segment depression and negative T-waves on the ECG. However, when qualification for emergency reperfusion therapy is based on ECG criteria, we lose approximately 25% of patients with acute TO of the IRA who do not present STE [2]. NSTEMI patients form a very heterogeneous group, and ESC guidelines recommend urgent coronary angiography only for those with life-threatening ventricular arrhythmias, resistant angina pectoris and haemodynamic instability. These conditions may be accompanied by total IRA occlusion, but this is not always the case. Thus, percutaneous coronary intervention (PCI) may be deferred in a substantial subset of NSTEMI patients with TO of the IRA, which may result in delayed myocardial salvage and poor cardiovascular outcomes [3].

This study aimed to identify the key points of clinical characteristics, course of treatment and outcome of patients with NSTEMI with TO of the IRA (NSTEMI_TO_) by comparison with the two most outlying groups: patients with NSTEMI and a non-occluded coronary artery (NSTEMI_NTO_) and patients with STE and an occluded IRA (STEMI_TO_).

## Methods

We analysed the data of patients with MI assembled within 48 months (2014–2017) into the Polish National Database of Invasive Coronary Procedures (ORPKI), coordinated by Jagiellonian University Medical College and endorsed by the Association of Cardiovascular Interventions of the Polish Cardiac Society [4]. All clinical data were collected by the operator and then uploaded into the database after each procedure. The diagnosis of NSTEMI or STEMI, recognition of the IRA, all clinical decisions during the coronary invasive procedure and definitions of periprocedural complications were left to the uploading ORPKI operators’ experience and knowledge according to current ESC guidelines.

Acute TO of the IRA was defined in our study as Thrombolysis In Myocardial Infarction (TIMI) 0 flow during coronary angiography in patients with MI [5].

To achieve the aim of the study, we compared 3 groups of patients: NSTEMI_TO_, NSTEMI_NTO_ and STEMI_TO_ and excluded from the analysis patients with STEMI and non-occluded coronary artery (STEMI_NTO_), those without significant coronary artery stenosis, those not treated with PCI and those diagnosed with chronic total occlusion of the IRA. To minimize confounding factors influencing the electrocardiographic presentation of MI, we decided to perform angiographic analysis only for patients with PCI of single native vessel disease.

Our study was an observational, non-experimental, retrospective analysis and was performed in accordance with the relevant guidelines and regulations. Only anonymized data were included in the research analysis, and according to Regulation 2016/679 of the European Parliament and of the Council (EU) from 27 April 2016 on the protection of individuals with regard to the processing of personal data and on the free movement of such data and with art. 9 section 2, this study did not require any additional ethics board approval. All subjects in our study gave informed consent for personal data processing by the Association of Cardiovascular Interventions of the Polish Cardiac Society before percutaneous coronary intervention.

### Statistical analysis

Categorical variables are presented as numbers and percentages. Continuous variables are expressed as the mean ± standard deviation (SD) or the median and interquartile range (IQR). The normality of continuous variables was assessed by the Kolmogorov–Smirnov–Lilliefors test. Equality of variance was assessed using Levene’s test. Differences between three groups were compared using classic one-way analysis of variance (ANOVA) or Welch’s ANOVA depending on the equality of variance for normally distributed variables. The Kruskal–Wallis test was used for ordinal or non-normally distributed continuous variables. Categorical variables were compared by Pearson’s chi-square test. All post hoc analyses were performed using the Benjamini–Hochberg procedure for controlling the false discovery rate (FDR). Two-sided p-values < 0.05 were considered statistically significant. All calculations were performed with JMP®, Version 14.2.0 (SAS Institute Inc., Cary, NC, USA).

## Results

**Fig. 1:**
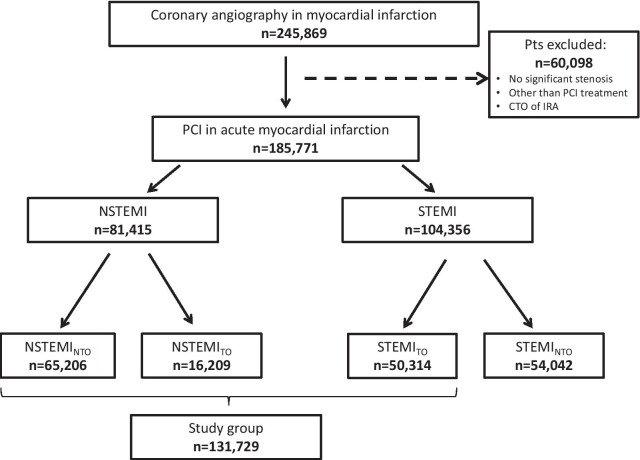
Study flowchart. PCI: percutaneous coronary intervention; CTO: chronic total occlusion; IRA: infarct-related artery; NSTEMI_NTO_: non-ST-segment elevation myocardial infarction without total occlusion of culprit artery; NSTEMI_TO_: non-ST-segment elevation myocardial infarction with total occlusion of culprit artery; STEMI_TO_: ST-segment elevation myocardial infarction with total occlusion of culprit artery; STEMI_NTO_: ST-segment elevation myocardial infarction without total occlusion of the culprit artery

The results of 245,869 coronary angiography procedures performed in patients with MI were entered into the ORPKI registry. After exclusion of patients (a) without significant stenosis of coronary arteries, (b) without occlusion of the IRA in STEMI, (c) without PCI treatment; (d) with chronic total occlusion of the IRA and (e) with multivessel PCI treatment, 131,729 patients who underwent single-vessel PCI constituted the study group. Among them, 65,206 (80.09%) patients with NSTEMI had no TO of the IRA (TIMI > 0), while total occlusion of the IRA (TIMI = 0) was found in 16,209 (19.91%) patients with NSTEMI and 50,314 (48.21%) with STEMI. The study flowchart is shown in Fig. 1.

### Clinical characteristics of the study groups

Patients with NSTEMI_TO_ were younger than those with NSTEMI_NTO_ but older than patients with STEMI_TO_. The percentage of smokers was highest in the STEMI_TO_ group, lower in the NSTEMI_TO_ group and lowest in NSTEMI_NTO_ group. The prevalence of chronic diseases (arterial hypertension, diabetes, chronic kidney disease, chronic obstructive pulmonary disease) was highest in the NSTEMI_NTO_ group, lower in the NSTEMI_TO_ group and lowest in the STEMI_TO_ group. All of the aforementioned differences were significant (p < 0.0001). A similar tendency was observed in the history of previous coronary revascularization (PCI or CABG), MI or stroke, which were most common in patients with NSTEMI_NTO_, less common in those with NSTEMI_TO_ and least common in those with STEMI_TO_ (p < 0.0001).

Clinical status on admission in the NSTEMI_TO_ group was more severe than that in the NSTEMI_NTO_ group but less serious than that in the STEMI_TO_ group. More advanced Killip classes were observed with the highest frequencies in patients with STEMI_TO_, lower frequencies in those with NSTEMI_TO_ and the lowest frequencies in those with NSTEMI_NTO_. Cardiac arrest before admission was more common in patients with STEMI_TO_ than in those with NSTEMI_TO_ and more common in patients with NSTEMI_TO_ than in those with NSTEMI_NTO_ (Table 1).

**Table 1:** Clinical characteristics of the study groups

	NSTEMI_NTO_ (N = 65,206)	NSTEMI_TO_ (N = 16,209)	STEMI_TO_ (N = 50,314)
Age (years), mean (SD)	68.78 (11.39)	65.98 (11.61)	64.86 (12.04)
Male sex^a^, n (%)	42.380 (65.19%)	11.215 (69.53%)*	34.133 (68.00%)
Weight (kg), mean (SD)	79.98 (17.49)	81.64 (17.54)	80.52 (16.67)
Smokers, n (%)	14.075 (21.59%)	4.303 (26.55%)	14.842 (29.50%)
Arterial hypertension, n (%)	47.872 (73.42%)	11.222 (69.23%)	29.912 (59.45%)
Diabetes, n (%)	17.774 (27.26%)	3.737 (23.06%)	8.729 (17.35%)
Kidney disease, n (%)	5.633 (8.64%)	948 (5.85%)	1.647 (3.27%)
COPD^b^, n (%)	1.718 (3.61%)	343 (2.90%)^$^	659 (1.75%)
Previous stroke, n (%)	2.877 (4.41%)	671 (4.14%)^#^	1.687 (3.35%)
Previous PCI, n (%)	17.433 (26.74%)	3.027 (18.67%)	6.010 (11.94%)
Previous CABG, n (%)	4.300 (6.59%)	835 (5.15%)	814 (1.62%)
Previous MI, n (%)	18.406 (28.23%)	3.550 (21.90%)	6.493 (12.90%)
Killip class III^c^, n (%)	1.051 (2.25%)	325 (2.47%)	1.431 (3.33%)
Killip class IV^c^, n (%)	787 (1.69%)	326 (2.48%)	2.162 (5.03%)
Cardiac arrest before admission^d^, n (%)	1.208 (2.19%)	477 (3.09%)	2.962 (6.02%)
Cardiac arrest during angiography^d^, n (%)	151 (0.27%)	82 (0.53%)	19 (0.04%)

p < 0.0001 for all analyses of the study groups by Kruskal–Wallis one-way analysis of variance

Data are presented as the mean and standard deviation (SD) or number (n) and percentage (%)

NSTEMI_NTO_: non-ST-segment elevation myocardial infarction without total occlusion of the culprit artery; NSTEMI_TO_: non-ST-segment elevation myocardial infarction with total occlusion of the culprit artery; STEMI_TO_: ST-segment elevation myocardial infarction with total occlusion of the culprit artery; COPD: chronic obstructive pulmonary disease; PCI: percutaneous coronary intervention; CABG: coronary artery bypass graft; MI: myocardial infarction

p < 0.0001 for all post hoc analyses with the following exceptions:

*p = 0.0003 for post hoc comparison between NSTEMI_TO_ and STEMI_TO_;

^$^p = 0.0002 for post hoc comparison between NSTEMI_NTO_ and NSTEMI_TO_;

^#^p = 0.1283 for post hoc comparison between NSTEMI_NTO_ and NSTEMI_TO_

Data available for ^a^ – 131,452 patients, ^b^ – 96,952 patients, ^c^ – 102,807 patients, ^d^ – 119,955 patients

### Time delays in MI treatment within study groups

**Fig. 2:**
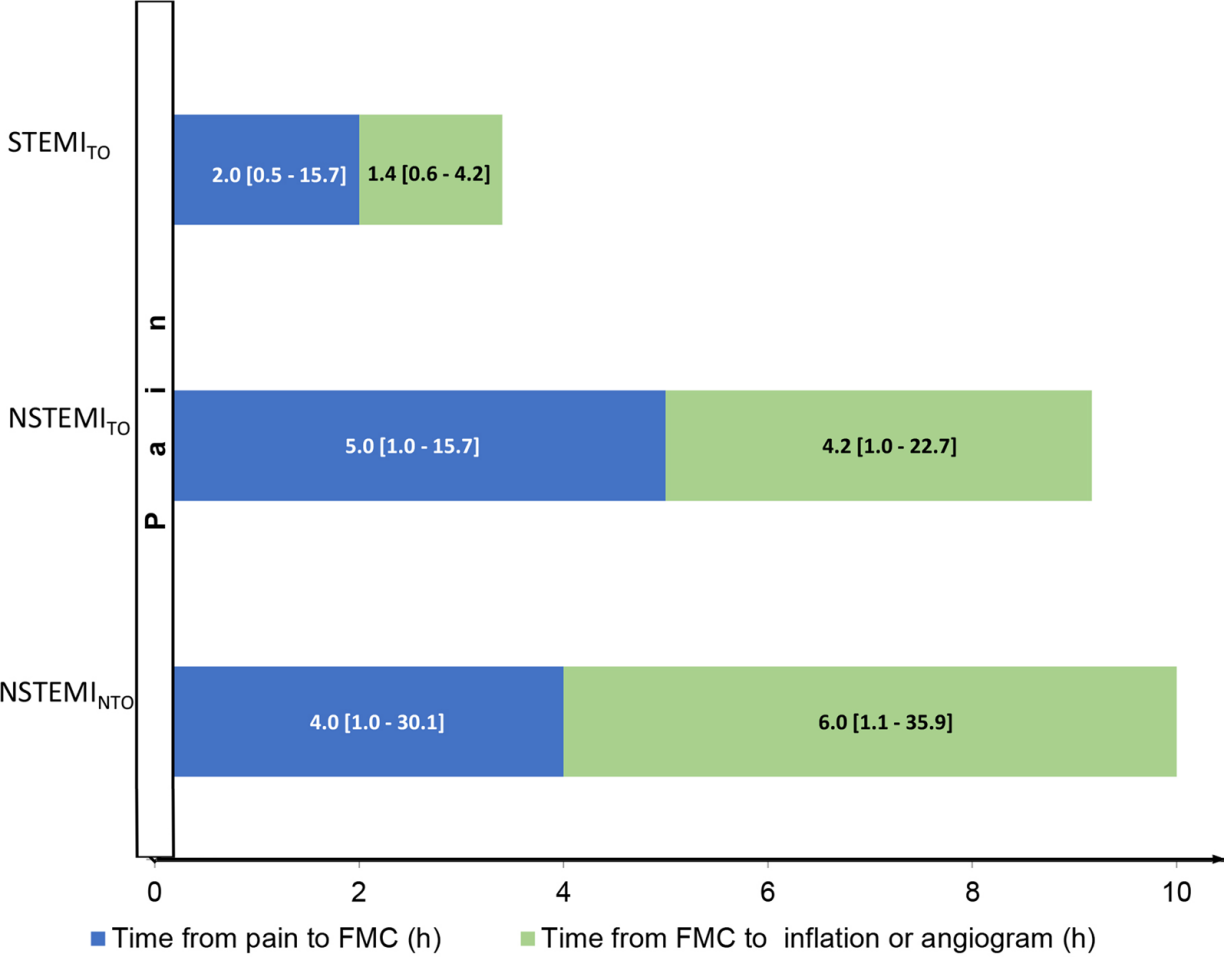
Comparison of median time from pain to first medical contact (FMC) and from FMC to balloon inflation or angiogram. Data are presented as the median, p < 0.0001 for all post hoc analyses

Direct transport to the catherization laboratory (Cath lab) was most common in the STEMI_TO_ group, less common in the NSTEMI_TO_ group and least common in the NSTEMI_NTO_ group. The time from pain to first medical contact (FMC) was longer in the NSTEMI_TO_ group than in either the STEMI_TO_ or NSTEMI_NTO_ group. Time periods (from pain to balloon inflation and from FMC to inflation) were shortest in the STEMI_TO_ group, intermediate in the NSTEMI_TO_ group and longest in the NSTEMI_NTO_ group. Time from FMC to inflation < 90 min and time from FMC to inflation < 120 min were observed most frequently in patients with STEMI_TO_, less frequently in those with NSTEMI_TO_ and least frequently in those with NSTEMI_NTO_ (Table 2, Fig. 2).

**Table 2:** Comparison of patient- and system-related delays to primary PCI

	NSTEMI_NTO_ (N = 65,206)	NSTEMI_TO_ (N = 16,209)	STEMI_TO_ (N = 50,314)
Direct transport to Cath lab^a^, n (%)	3.682 (6.66%)	1.412 (9.14%)	1.2645 (25.69%)
Time from			
Pain to FMC^b^ (h), median (IQR)	4.00 (2.00–11.00)	5.00 (2.00–14.00)	2.00 (1.00–5.53)
Pain to inflation^c^ (h), median (IQR)	14.42 (7.00–30.98)	12.48 (6.38–27.00)	4.00 (2.33–8.50)
FMC to inflation^d^ (h), median (IQR)	6.00 (2.42–17.00)	4.17 (2.00–9.67)	1.40 (0.97–2.25)
FMC to inflation^d^ < 90 min, n (%)	7.008 (14.05)	2.618 (18.61)	24.268 (53.07)
FMC to inflation^d^ ≤ 120 min, n (%)	9.553 (19.16)	3.445 (24.48)	31.098 (68.01)

p < 0.0001 for all analyses of the study groups by Kruskal–Wallis one-way analysis of variance

Data are presented as the median and interquartile range (IQR) or number (n) and percentage (%);

NSTEMI_NTO_: non-ST-segment elevation myocardial infarction without total occlusion of culprit artery; NSTEMI_TO_: non-ST-segment elevation myocardial infarction with total occlusion of culprit artery; STEMI_TO_: ST- segment elevation myocardial infarction with total occlusion of culprit artery; Cath lab: catheterization laboratory;

p < 0.0001 for all post hoc analyses;

Data available for ^a^ – 119,955 patients, ^b^ – 107,435 patients, ^c^ – 109,566 patients, ^d^ – 109,664 patients

### Results of coronary angiography

**Fig. 3:**
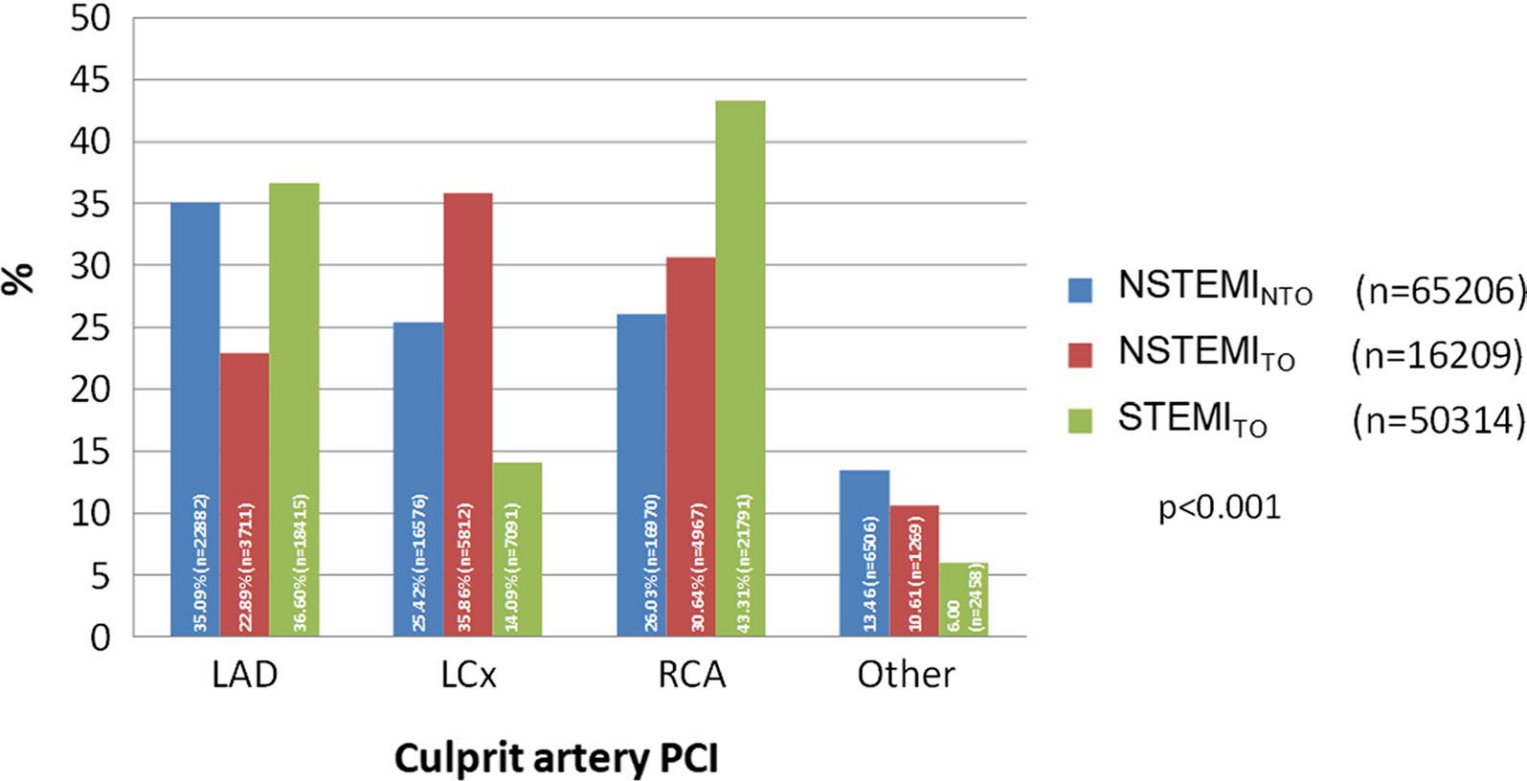
The frequency rate of culprit lesions for the left anterior descending artery (LAD), left circumflex artery (LCx), right coronary artery (RCA) and other arteries within the study groups. p < 0.0001 for all analyses of study groups by Kruskal–Wallis one-way analysis of variance, p < 0.0001 for all post hoc analyses, p for contingency analysis < 0.0001

In our study, the left anterior descending artery (LAD) was identified as the IRA in 45,008 patients; the left circumflex artery (LCx), in 29,479 patients; and the right coronary artery (RCA), in 36,828 patients. Other culprit localizations (i.e., left main, coronary artery bypass grafts, bifurcations) were found in 10,223 patients, and this subset of patients who did not fulfil the criteria of single native vessel disease PCI was excluded from further analysis (Fig. 3). Among patients with NSTEMI_TO_, LCx was the most frequent single native vessel occlusion. In contrast, occlusion of LAD as the culprit lesion was observed the least often in this group. In patients with STEMI_TO_, LCx occlusion was infrequent, while occlusion of the RCA or LAD was prevalent. NSTEMI_NTO_ was related most often to LAD as the culprit lesion, less commonly to RCA and least often to LCx (p for contingency analysis < 0.0001).

### Analyses of PCI results

The successful revascularization outcome defined as TIMI flow grade after PCI in the NSTEMI_TO_ group was worse than that in the STEMI_TO_ and NSTEMI_NTO_ groups (Table 3). TIMI flow grade 3 was reached with the lowest frequency in the IRA after PCI, and TIMI flow grade 0 after PCI was noticed with the highest occurrence rate in the NSTEMI_TO_ group compared with both the STEMI_TO_ and NSTEMI_NTO_ groups. The no-reflow phenomenon, cardiac arrest during PCI and death during the invasive procedure in the NSTEMI_TO_ group occurred less frequently than in the STEMI_TO_ group but more frequently than in the NSTEMI_NTO_ group.

**Table 3:** Percutaneous coronary intervention results

	NSTEMI_NTO_ (N = 65,206)	NSTEMI_TO_ (N = 16,209)	STEMI_TO_ (N = 50,314)
TIMI 3 after PCI^a^, n (%)	62.114 (95.57)	13.483 (83.36)*	44.494 (88.61)
TIMI 2 after PCI^a^, n (%)	1.678 (2.58)	834 (5.16)*	2.921 (5.82)
TIMI 1 after PCI^a^, n (%)	531 (0.82)	333 (2.06)*	971 (1.93)
TIMI 0 after PCI^a^, n (%)	672 (1.03)	1.525 (9.43)*	829 (3.64)
No reflow after PCI, n (%)	315 (0.48)	218 (1.34%)*	878 (1.75%)
Cardiac arrest during PCI, n (%)	361 (0.55)	175 (1.08)*	1.198 (2.38%)
Death during PCI, n (%)	281 (0.43)	158 (0.97)*	885 (1.76%)

p < 0.0001 for all analyses of study groups by analysis of variance

NSTEMI_NTO_: non-ST-segment elevation myocardial infarction without total occlusion of culprit artery; NSTEMI_TO_: non-ST-segment elevation myocardial infarction with total occlusion of culprit artery; STEMI_TO_: ST- segment elevation myocardial infarction with total occlusion of culprit artery; TIMI: Thrombolysis in Myocardial Infarction; PCI: percutaneous coronary intervention

p < 0.0001 for post hoc all analyses with the following exception: *p = 0.0005 for post hoc comparison between NSTEMI_TO_ and STEMI_TO_; ^a^ – data available for 131,385 patients

## Discussion

To the best of our knowledge, we conducted the largest single-study analysis dedicated to the NSTEMI_TO_ phenomenon (16,209 patients). The previous meta-analyses on this topic included 10,415 patients (7 studies) and 17,212 patients (25 studies) with NSTEMI_TO_ [6, 7].

Our study results suggest that NSTEMI_TO_ may be considered an intermediate condition between NSTEMI_NTO_ and STEMI_TO_. However, the following features make the NSTEMI_TO_ group exceptional:

- The longest time delay to obtain proper medical care (patients with NSTEMI_TO_ reached FMC when STEMI_TO_ patients had already undergone PCI),
- LCx as the most frequent infarct-related artery, and
- The worst final result of PCI.

Numerous studies have shown the differences in the baseline clinical presentation between patients with STEMI and NSTEMI. In the OPERA Registry, correlates of mid- and long-term mortality were similar for NSTEMI and STEMI patients [8]. This leads to the conclusion that we should not consider STEMI and NSTEMI to be two different diseases but rather as an ischaemic continuum due to subtotal or total occlusion of the coronary artery with different ECG manifestations [9, 10]. Total occlusion of the IRA can occur in STEMI and NSTEMI patients. Numerous studies have compared acute total occlusion of the IRA with non-total occlusion of the IRA but mostly within the NSTEMI subset of patients [11, 12]. Our goal was to compare three manifestations of acute MI: NSTEMI_NTO_, NSTEMI_TO_ and STEMI_TO_; thus, for the first time, we compared three groups instead of two.

Considering the baseline characteristics, patients with NSTEMI_TO_ in our study constituted an intermediate group between NSTEMI_NTO_ and STEMI_TO_. In comparison to STEMI_TO_ patients, NSTEMI_TO_ patients were older and had a higher prevalence of cardiovascular risk factors and chronic diseases. When comparing NSTEMI_TO_ to NSTEMI_NTO_, the patients were younger and had a lower prevalence of cardiovascular risk factors and chronic diseases. These findings are in accordance with other studies, where patients with NSTEMI, in comparison to STEMI, were older and more often had chronic diseases [13, 14]. According to the baseline characteristics, our NSTEMI_TO_ group was definitely closer to the STEMI_TO_ group than to the NSTEMI_NTO_ group. Patients with STEMI_NTO_ were excluded due to high group heterogeneity. To summarize the results of pre-hospital management, participants with NSTEMI_TO_ were generally less frequently considered candidates for direct transportation to the Cath lab than STEMI_TO_ patients (9.41% vs 25.69%). Additionally, ischaemia time, i.e., time from pain to balloon inflation, as well as time from FMC to balloon inflation, were longer in the NSTEMI_TO_ group than in the STEMI_TO_ group. The duration of ischaemia is a major determinant of infarct size and subsequent mortality [3, 15]. In almost all studies included in the meta-analysis of Khan et al., patients with NSTEMI_TO_ had a mean delay to invasive procedure longer than 24 h and, in comparison to patients with NSTEMI_NTO_, an increased risk of both major adverse cardiovascular events and mortality [6]. The mean time from pain to inflation in our study was approximately 30 h (data not presented) and was similar to that presented by Khan et al. [6].

The time from pain to FMC was the longest in the NSTEMI_TO_ group and was even longer than that in the NSTEMI_NTO_ group. In the NSTEMI_TO_ group, patients postponed the decision to seek medical help, probably because of younger age (than in the NSTEMI_NTO_ group) and a lack of previous experience with stenocardial pain. The longer time delay from pain to FMC in NSTEMI_TO_ than in STEMI_TO_ may be explained by the lower severity of symptoms due to the lower degree of ischaemia in the case of LCx occlusion.

The time delay to achieve the opening of the occluded artery in the NSTEMI_TO_ group in comparison to the STEMI_TO_ group was amplified during in-hospital management, which was noticeable as the pronounced difference (median time from FMC to balloon inflation was almost three times longer in the NSTEMI_TO_ group).

In contrast, patients with NSTEMI_TO_ in comparison to those with NSTEMI_NTO_ were previously considered candidates for invasive management. The potential explanation is the more severe clinical presentation caused by total artery occlusion. The higher frequency of cardiac arrest before admission and more advanced Killip class in the NSTEMI_TO_ group than in the NSTEMI_NTO_ group in our study confirm this hypothesis. Similar results were obtained by Shin et al. in the COREA‐AMI Registry [16]. Another commonly used parameter of time delay in MI that influences prognosis is the percentage of patients who undergo PCI within 120 min since the onset of symptoms [3, 15, 17]. In the study of Terkelsen et al., approximately 50% of STEMI patients had balloon inflation within 120 min [17]. In our study, almost 70% of STEMI_TO_ patients, but only 25% of NSTEMI_TO_ patients, had PCI within 120 min.

Approximately 20% of our NSTEMI patients had acute coronary artery occlusion, which is less than that previously reported by Khan (25.5%) and Hung (34%) [6, 7]. This difference may be explained by the fact that we used a stricter definition of NSTEMI_TO_, analysing only patients with TIMI 0 flow, whereas Khan and Hung included patients with TIMI 0–1. Previous studies examining the distribution of occluded arteries in NSTEMI_TO_ patients indicated that the RCA or LCx was the artery most responsible for NSTEMI_TO_ [6, 7]. In our study, we found that the LCx was the most typical localization of the culprit lesion responsible for MI in the NSTEMI_TO_ group. The distribution of the IRA differs between trials when STEMI cases are compared to NSTEMI, i.e., in STEMI, there is underrepresentation of the LCx as the IRA [18], whereas in NSTEMI_TO,_ occlusion of the LAD occurs the least often [7, 19]. We must acknowledge that ECG has unsatisfactory sensitivity for diagnosis of coronary artery total occlusion, especially in posterolateral distribution [20].

In our study, patients with NSTEMI_TO_ demonstrated a more severe clinical condition on admission than those with NSTEMI_NTO_ (more advanced Killip class, higher prevalence of death and cardiac arrest prior to admission or during an invasive procedure, no-reflow phenomenon), which is in concordance with prior studies showing that the prognosis of patients with total occlusion without ST-segment elevation is worse than that of NSTEMI_NTO_ patients [6, 7]. We confirmed that the outcome after PCI (lower frequency of achieving TIMI 3 and higher frequency of TIMI 0) in NSTEMI_TO_ patients is even inferior to that in STEMI_TO_ patients. A possible explanation is that unrecognized acute coronary artery occlusion is associated with high morbidity and mortality [15], and the outcome in this group is worse than that in the group that received timely revascularization [11, 21].

In summary, we must acknowledge that the identification of NSTEMI_TO_ patients prior to coronary angiography remains challenging. Among the NSTEMI patients in our study, younger patients with a lower frequency of comorbidities (which is not a typical NSTEMI group characteristic) should be highly suspected of having acute total occlusion of the infarct-related artery if they present severe symptoms on admission (i.e., advanced Killip class, pre-hospital cardiac arrest, long pain duration), which enables us classify them into the very high-risk group according to the most current 2020 ESC NSTEMI guidelines [22]. To select high-risk patients who require urgent coronary intervention, we should exert additional effort and use all available methods, i.e., careful clinical assessment, ECG analysis including additional ECG leads (i.e., V7-V9) and patterns that are highly suggestive of TO of the IRA (extensively described in the supplementary table of 2020 ESC NSTEMI guidelines), specific risk score calculation and echocardiography examination with wall motion and strain analysis [22].

### Study limitations

Our study has several limitations. First, we should be very cautious about drawing conclusions about detailed in-hospital prognoses because our analysis is based on data from the structured registry that included prespecified clinical and a periprocedural data spectrum only, without longitudinal follow-up, but with the largest number of evaluated patients thus far. Second, the registry was created, and data were entered by several operators; the quality of data depends on their individual knowledge. However, only the most experienced operators collected the data.

## Conclusions

Approximately one-fifth of NSTEMI patients had acute total coronary artery occlusion (NSTEMI_TO_). According to the clinical characteristics, NSTEMI_TO_ seems to be an intermediate condition between NSTEMI_NTO_ and STEMI_TO_. However, it should be emphasized that NSTEMI_TO_ patients have the longest time delay to PCI and the worst final result of PCI, which can be at least partially explained by the most common location of the culprit lesion in the LCx. Therefore, patients with NSTEMI should undergo strict evaluation for signs indicating possible acute total coronary artery occlusion (e.g., younger age, lower cardiovascular risk, fewer chronic diseases but more severe clinical presentation on admission) and should undergo comprehensive ECG and echocardiographic assessment to prevent a time delay to and improve the results of revascularization.

## Data Availability

The datasets used and/or analysed during the current study are publicly available from the Jagiellonian University Medical College and the Association of Cardiovascular Interventions of the Polish Cardiac Society.

## Abbreviations

CABG: Coronary artery bypass graft
COPD: Chronic obstructive pulmonary disease
CTO: Chronic total occlusion
ECG: Electrocardiogram
ESC: European Society of Cardiology
FMC: First medical contact
IRA: Infarct-related artery
LAD: Left anterior descending artery
LCx: Left circumflex artery
MI: Myocardial infarction
NSTEMI_NTO_: Non-ST-segment elevation myocardial infarction without occluded infarct-related artery
NSTEMI_TO_: Non-ST-segment elevation myocardial infarction with occluded infarct-related artery
ORPKI: Polish National Database of Invasive Coronary Procedures
PCI: Percutaneous coronary intervention
RCA: Right coronary artery
STE: ST-segment elevation
STEMI_TO_: ST-segment elevation myocardial infarction with occluded infarct-related artery
TIMI: Thrombolysis in myocardial infarction
TO: Total occlusion
